# Roles of efferocytosis in wound repair: Process, cells, and signals

**DOI:** 10.1016/j.gendis.2025.101937

**Published:** 2025-11-13

**Authors:** Yilin Sun, Haiying Guo, Yang Bai, Jin Chen, Yuhong Li

**Affiliations:** a Department of Cell Biology, Army Medical University, Chongqing 400038, China; b Department of Otolaryngology, The First Affiliated Hospital, Army Medical University, Chongqing 400038, China; c Institute of Clinical Medicine, The Second Affiliated Hospital of Hainan Medical University, Haikou, Hainan 570311, China

**Keywords:** Cells, Efferocytosis, Inflammation, Signals, Wound repair

## Abstract

Efficient clearance of apoptotic cells, termed efferocytosis, is essential for resolving excessive inflammation, promoting wound repair, and maintaining homeostasis. Defective clearance results in the accumulation of dead cells and other metabolites, which are responsible for chronic inflammation, nonhealing of wounds, and tissue regeneration. Emerging evidence shows that the failure to resolve inflammation and defective phagocytosis or efferocytosis increases the possibility of several diseases involving diabetic wounds and damage to the gastrointestinal mucosa in patients with inflammatory bowel disease, which is a focus of medical development and the public eye. Thus, gaining deeper insight into the molecular and cellular mechanisms of efferocytosis may be useful for inflammation resolution. This review describes the mechanism of efferocytosis and wound repair and the roles of professionals (macrophages and dendritic cells) and amateur phagocytes (*e.g.*, epithelial cells, endothelial cells, and fibroblasts) in both processes, which may provide insight into how efferocytosis affects wound repair. Because there may be many inflammatory cells recruited to the injury area, the aim of efferocytosis is to clear these cells and release proinflammatory and anti-inflammatory mediators to promote repair. Here, we review the effects of cell-mediated efferocytosis on the timely efferocytosis of neutrophils and M1 macrophages and the relationship between M2 polarization and efferocytosis. In addition, the molecular mechanisms involved are discussed, which may further our understanding of the effects of efferocytosis. Finally, these signals also provide potential targets for tissue repair intervention.

## Introduction

During development, stress, and infection, approximately 0.4% of the estimated 37.2 trillion cells die every day.^1^^,^^2^ Apoptosis is important to normal organ development and tissue homeostasis. This process usually does not come with inflammation due to the efficient efferocytosis.^3^ The clearance of apoptotic cells by professional macrophages and dendritic cells and amateur phagocytes^1^ (*e.g.*, epithelial cells, endothelial cells, and fibroblasts, named efferocytosis) is pivotal for the maintenance of tissue repair and homeostasis.^4^ Consecutive and well-organized efferocytosis processes degrade apoptotic cells and cellular debris in a non-phlogistic manner, which involves migration toward apoptotic cells, recognition and phagocytosis of apoptotic cells, and their internalization.^2^^,^^5^

Currently, efferocytosis is a crucial factor in various disease models, including cardiac repair after atherosclerosis, skeletal muscle repair, diabetic wound healing, and gastrointestinal mucosa repair in inflammatory bowel disease.^2^ For example, in 2021, 529 million people were living with diabetes worldwide, and many of them may suffer from diabetic foot ulcers, which are associated with a high risk of limb loss through amputation.^6^^,^^7^ Atherosclerosis is a disease characterized by decreased efferocytosis of apoptotic bodies. It is a common underlying cause of nonhealing skin wounds of the lower leg.^8^ Moreover, these diseases may evolve into a chronic wound defined as a barrier defect that has not healed in 3 months, which has become one of the events that annoyed Western countries.^9^ These examples are all related to ineffective efferocytosis.

The effective clearance of apoptotic cells is crucial for wound resolution and tissue reconstruction. Impeded clearance can lead to a protracted inflammatory environment that further affects wound healing.^10^ Therefore, it is important to utilize efferocytosis to promote the repair of the above wounds. Cell-related efferocytosis, as well as some important pathways or cytokines involved in efferocytosis, can be activated or inhibited to promote wound healing. Previous reviews mostly focused on the role of macrophages or neutrophils in tissue repair. We highlight the contributions of different cell types involved in the efferocytosis process in wound repair, not only through the use of professional phagocytes but also through the use of other nonprofessional phagocytes and inflammatory cells. Finally, we introduce newer approaches and research directions that have the potential to further our understanding of the mechanisms underlying tissue repair.

## The process of efferocytosis

Efferocytosis is a carefully orchestrated process that can be generally categorized into three steps: i) the recruitment of phagocytes via “find-me” signals; ii) the recognition and engagement of “eat-me” and “don't eat-me” signals; iii) the engulfment, processing, and degradation of the cellular corpse, as well as the immune response to the engulfed corpse.^2^

These signals involve both feed-forward and feedback regulation. For instance, downstream molecules, such as proinflammatory cytokines (TNF-α, IL-6, and IL-1β), are not properly ingested, resulting in defective efferocytosis, which may result in trauma repair disorders.^11^

## “Find-me” stage

In the first stage, apoptotic cells must send signals termed “find-me” signals to stimulate the migration of phagocytes to the location of cell death. The most well-known “find-me” signals are nucleotides, such as uridine triphosphate and adenosine triphosphate, which are released by cell corpses, and lipopolysaccharide (LPS), which is derived from bacteria, can act as damage-associated molecular patterns (DAMPs) or pathogen-associated molecular patterns (PAMPs).^12^ Cell debris releases these nucleotides into the environment through the plasma membrane channel pannexin-1.^13^^,^^14^ Caspase-3 and caspase-7 play important roles in activating pannexin-1.^13^ The connection between these nucleotides and the P2X7 receptor or P2Y receptor (a family of purinergic G protein-coupled receptors located in resident macrophages, infiltrating neutrophils, monocytes, or dendritic cells) may lead to the influx of calcium ions.^12^ This process can be related to hemocyte recruitment for the production of hydrogen peroxide at wound sites via the NADPH oxidase Duox with a rapid calcium flash in worms, flies, and fish.^15^

S100A8/SA00A9, which can be released from adipose tissue, can interact with the toll-like receptor (TLR4) localized on the plasma membrane and in intracellular endosomes of phagocytes and activate the NLR family as well as protease caspase-1, which processes cytokines such as interleukin-1β (IL-1β).^16^^,^^17^ TLR engagement primes neutrophils for enhanced responses to other stimuli, thus augmenting their phagocytic capacity and stimulating increased cytokine release.^18^ Defective TLR signals may lead to unpaired wounds in diabetes patients.^19^ Nuclear factor-kappa B (NF-κB) ubiquitously acts as a heterodimer of the p50 and p65 subunits. The synthesis of inhibitor kappa B alpha (IκBα), which is dependent on the p65 subunit, inhibits the transcription factor activity of NF-κB.^20^ NF-κB signaling can be activated by stimulants such as TLRs and then up-regulate the expression of inflammatory cytokines involving tumor necrosis factor alpha (TNF-α) in the inflammatory phase of wound closure, where TLR2 and TLR4 recognize microbial infection via the IkBα of NF-κB through the adaptor molecule MyD88. This can also explain the polarization of M2 macrophages and one of the mechanisms by which statins promote cardiac repair after atherosclerosis.^21^^,^^22^ The binding of lysophosphatidylcholine (LPC) to the G-protein-coupled receptor (G2A) on macrophages and monocytes through ATP-binding cassette transporter A1 (ABCA1) promotes the migration and phagocytosis of apoptotic cells. Knockdown of ABCA1^23^ and G2A^24^ can reduce both the migration and engulfment of phagocytes (Table 1).

**Table 1:** Stages of efferocytosis in wound repair.

Stage	Molecular mechanism	Key molecules/receptors	Representative references	Effect
Find-me	Apoptotic cells release chemotactic signals	CXCL1, ATP, S1P	62	Recruit phagocytes to the apoptotic site
Eat-me	Surface exposure “eat-me” signals	Phosphatidylserine (PS), lysophosphatidylcholine (LPC), calreticulin	63	Trigger phagocytosis recognition receptors
Engulfment	Cytoskeleton remodeled to form a phagocytic cup	Rac1/CDC42, ELMO-DOCK180	64	Complete endocytosis
Digestion	Lysosome fusion and anti-inflammatory factor release	TGF-β, IL-10	65	Inflammation subsides/Tissue regeneration priming

**Figure 1:**
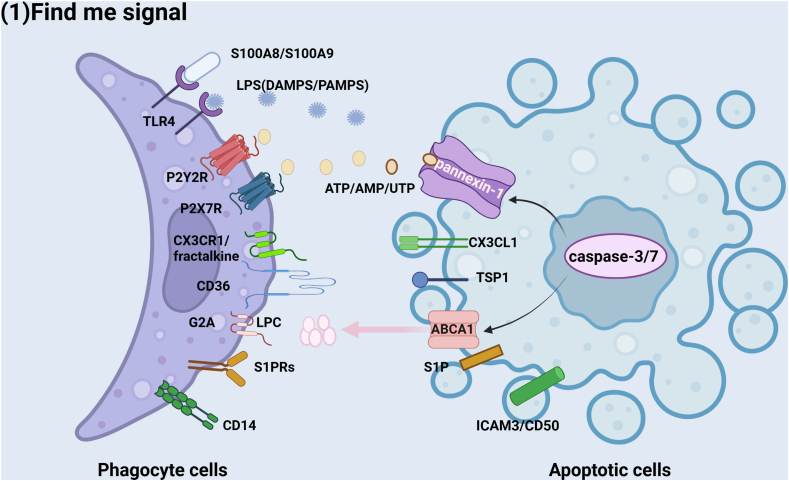
Mechanisms of signaling in efferocytosis. “Find-me” signals: Apoptotic cells send signals termed “find-me” signals, such as ATP/UTP/AMP, LPS, and LPC, to stimulate the migration of phagocytes, and the receptors on phagocytes can bind to these signals, which provides a way to eat these apoptotic cells. These processes may involve the participation of channel proteins, such as ABCA1 and pannexin-1.

Sphingosine-1-phosphate (S1P) is responsible for the recruitment of phagocytes to apoptotic cells. The release of S1P from apoptotic cells helps promote macrophage polarization by inducing the up-regulation of peroxisome proliferator-activated receptor-γ (PPAR-γ), thereby increasing the production of efferocytic surface bridging molecules.^19^ PPAR-γ can also be activated by the injection of phosphatidylserine (PS) in chronic granulomatous disease, an inflammatory disease.^25^ Apoptotic cells also release the classical chemokine membrane-associated molecule C-X3-C motif chemokine ligand 1 (CX3CL1) (or fractalkine) in small vesicles or microparticles, which can be sensed by CX3CR1 on phagocytes (Fig. 1)^2^

## “Eat-me” and “don't eat-me” stage

To date, many “eat-me” ligands with different flavors, including PS, carbohydrates (*e.g.*, amino sugars and mannose), and calreticulin, have been identified.^26^ The key trigger is PS, as PS can be recognized directly by apoptotic cell-specific receptors or recognized and bound by bridging molecules.^27^^,^^28^ After cell death, PS transits from the cytoplasmic leaflet to the exoplasmic leaflet with the help of flippase (a P-type ATPase, ATP11C) via the scramblase Xk-related protein 8 (Xkr8) to maintain its imbalanced distribution on the membrane.^19^ Annexin A1 expressed on the plasma membrane can colocalize with PS because it transforms from the inner to the outer membrane and significantly stimulates the engulfment of apoptotic cells.^13^ Annexin A1 plays an important role in increased efferocytosis and antigen-presenting machinery ability in dendritic cells.^29^ Stabilin-1, stabilin-2, T-cell immunoglobulin mucin receptor-1/4 (TIM1/4), brain-specific angiogenesis inhibitor 1 (BAI1), CD300b, and RAGE can bind to PS directly. Although they share different sites of action from PS, they all activate downstream molecules and promote anti-inflammatory processes.^26^ Park et al demonstrated that stabilin-1 derived from bone marrow-derived macrophages mediates the engulfment of apoptotic cells in a PS-dependent manner.^30^ Stabilin-2, a membrane receptor, engulfs apoptotic cells in a PS-specific manner essential for resolving inflammation and increases the production of anti-inflammatory cytokines such as transforming growth factor beta (TGF-β) by human monocyte-derived macrophages.^19^^,^^31^ TIM-4 is also considered to be a PS receptor that is directly involved in the recognition of apoptotic bodies.^32^ We can use p38 inhibitor-dependent mitogen-activated protein kinase (MAPK) signaling to activate TIM-4 expression to enhance the ability to clear apoptotic bodies.^33^ BAI1 is another PS-binding receptor whose cytoplasmic tail domain is among the earliest identified direct receptors in primary fibroblasts.^28^^,^^34^^,^^35^ Additionally, functional experiments have confirmed that BAI1 forms a trimeric complex with engulfment and cell motility (ELMO) and dedicator of cytokinesis (DOCK180) to promote cytoskeletal reorganization and the maximal number of phagocytes during efferocytosis.^34^^,^^35^ Murakami and colleagues reported that CD300b could recognize PS in the absence of TIM1 or TIM4. Both the inhibition of CD300b and siRNA transfection could decrease the phagocytosis of apoptotic cells.^36^ It has been reported that advanced glycation end products (RAGE) can participate in the “eat-me” signal as a ligand of PS by He and colleagues. RAGE-deficient (*Rage*^*−/−*^) alveolar macrophages impair phagocytosis in diabetes and affect the activation of the downstream molecule Rac family small GTPase 1 (Rac1).^26^^,^^37^ The above protein is directly bound to PS, and some indirectly identified proteins are described next. The TAM receptor family, composed of three receptor tyrosine kinases, Axl, Tyro3, and MerTK, binds to PS on apoptotic cell membranes via Gas6 and Protein S as bridging molecules.^38^^,^^39^ Both Gas6 and Protein S are vitamin K-dependent proteins that activate efferocytosis in a Ca^2+^-dependent manner.^13^ To confirm the association between MerTK and Gas6, human macrophages incubated with the endogenous MerTK ligand Gas6 were used, and then, the levels of SPM (a proresolving mediator of inflammation) in both *Mertk*^*−/−*^ and wild-type macrophages were analyzed, and the results revealed a decrease in *Mertk*^*−/−*^ macrophages.^40^ Combined deficiency of MerTK and Axl was shown to restrain macrophage proliferation in worm-infected mice, indicating decreased efferocytosis and wound repair.^41^ Mice infected with Brasiliensis usually suffer lung tissue damage. Red blood cell numbers in the bronchoalveolar lavage fluid were markedly reduced by day 4 and returned to uninfected baseline levels by day 7 in wild-type mice, while *Axl*^*−/−*^*Mertk*^*−/−*^ mice continued to bleed even on day 7.^42^ Moreover, Mertk deficiency is linked to increased infarct size in atherosclerosis as well as a lupus-like phenotype in aged mice because of defective clearance of cell debris.^40^^,^^43^ Milk fat globule-epidermal growth factor (EGF)-factor VIII (MFG-E8), which contains two EGF domains, is a glycoprotein secreted by MΦ and dendritic cells. While engaged by PS on apoptotic cells, MFG-E8 is more likely to bind to dying cells with integrins αvβ3 and αvβ5 via its arginine-glycine-aspartate (RGD) motif.^44^, ^45^, ^46^ There are other receptors in addition to PS, such as calreticulin and oxidized low-density lipoproteins, on apoptotic cells. CD36, a membrane glycoprotein present on various kinds of cells, binds to low-density lipoproteins in the process of phagocytosis.^5^ Calreticulin plays a key role as a bridging molecule in recognizing and engulfing apoptotic cells via low-density lipoprotein receptor related proteins 1 (LRP1) signaling to CD91 by directly binding to the complement protein component 1q (C1q), which has been shown to be involved in a range of cell processes, such as differentiation, chemotaxis, aggregation, and adhesion, during efferocytosis (Table 1).^13^^,^^47^^,^^48^

**Figure 2:**
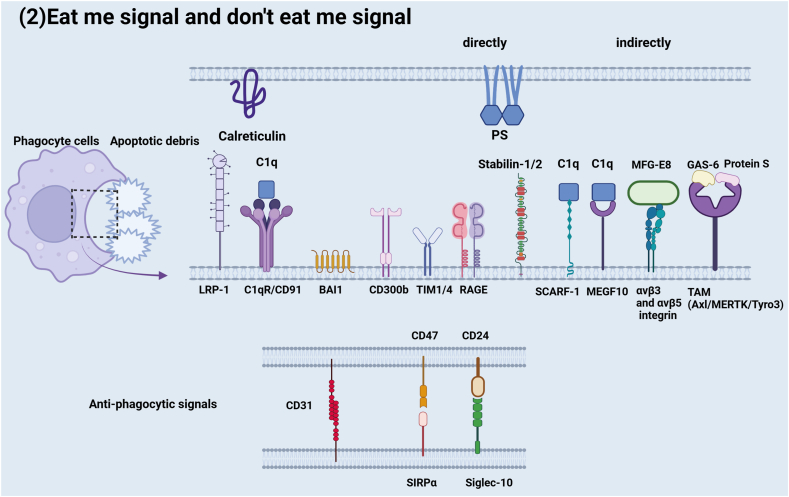
Mechanisms of signaling in efferocytosis. There is a balanced mechanism between “eat-me” and “don't eat-me”. The most familiar ligand is phosphatidylserine (PS), which can directly or indirectly bind to receptors on phagocyte cells. For example, PS can bind to CD300b and BAI1 without bridging molecules, while PS acts on TAM receptors with the help of protein S and GAS-6.

Only the “eat-me” signal is not enough. Some markers upgraded on the surface of nonapoptotic cells can be protected from engulfment by phagocytic cells, resulting in a critical balance between eating me and not eating me in the body. CD47 is usually expressed on normal cells and prevents untimely apoptosis. CD47 is correlated with signal regulatory protein alpha (SIRPα), a protein chiefly expressed on macrophages and dendritic cells.^49^ CD47 activates SIRPα by inhibiting downstream signaling via its ITIM motifs in the extracellular region.^50^^,^^51^ It has been revealed that using anti-SIRPα blocking antibodies results in senescent cell-mediated efferocytosis suppression.^52^ An anti-CD47 antibody suppresses intraplaque Src homology region 2 (SH-2) domain-containing phosphatase 1 (SHP1)/SHP2 phosphorylation, which decreases the interaction with SIRPα.^53^^,^^54^ The anti-CD47 antibody can also be applied to autoimmune joint inflammation, inflammatory colitis, and atherosclerosis by reducing neutrophils and monocytes in tissues through the reactivation of efferocytosis.^54^ It was reported that CD47-SIRPα inhibits the inside-out activation of integrin to reduce efferocytosis.^55^ Barkal et al have confirmed that CD24–Siglec-10 signaling is involved in the anti-tumor immune response via the recognition of tumor cells by macrophages. The use of Siglec-10 monoclonal antibodies increases the engulfment ability of macrophages, revealing a role for Siglec-10 in reducing efferocytosis (Fig. 2).^56^

## “Engulfment and digestion” stage

**Figure 3:**
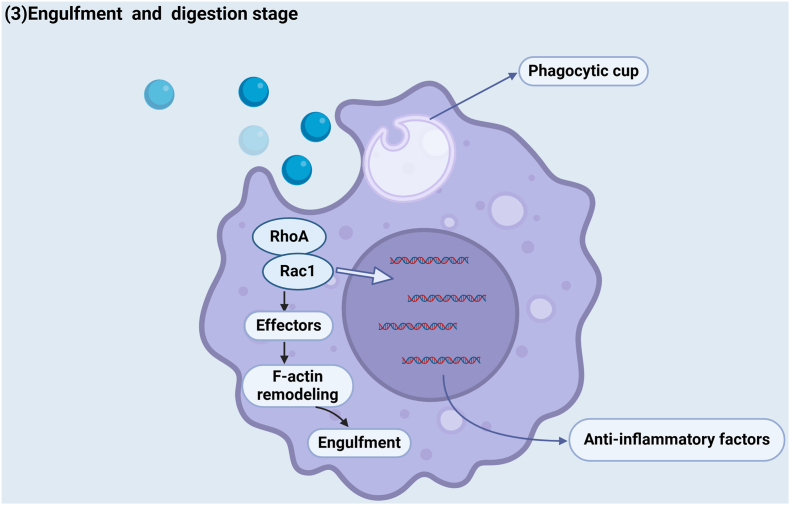
Mechanism of signaling in the postengulfment and digestion stage and the release of anti-inflammatory factors. The engulfment and digestion refer to the formation of phagolysosomes, which can engulf internalized corpses via energy conversion, leading to the activation and release of anti-inflammatory mediators. This process inhibits the excessive inflammation of cells and even necrosis.

The last stage involves processing of the corpses and the production of anti-inflammatory mediators, including prostaglandin E2 (PGE2), TGF-β, IL-10, and lactate.^11^ During this step, Rho family GTPases, such as Rac1 and cell division cycle 42 (Cdc42), regulate the cytoskeleton to form a phagocytic cup for further phagocytosis and degradation.^57^^,^^58^ The formation and activation of a phagocytic cup also need to be precisely regulated to prevent the phagocytosis and digestion of phagocytic cells from being too strong or too weak. The “phagocytic cup”-mediated internal phagocyte process differs from traditional immunological recognition, which is balanced by GTPase and can be revealed in *C. elegans*, Drosophila, and mammals.^59^ The phagocytic cup formation process is as follows. Once corpses are internalized, the phagosome vacuole can mix with primary lysosomes or the product of the endoplasmic reticulum or Golgi complex to form a secondary phagolysosome. This process is dynamic in that it involves the fusion of endocytic and secretory vesicles, resulting in the simultaneous digestion of cargo and extensive membrane recycling.^60^ After the fusion of late phagosomes and lysosomes, there may be a series of downstream mechanisms to adjust the digestion of phagocytic cell corpses and cytoskeleton remodeling. Rac-1, a positive regulator of efferocytosis, and RhoA, a negative regulator of efferocytosis, indicate that statins such as lovastatin have positive effects on efferocytosis via RhoA inhibition. Cardiac repair after atherosclerosis largely relies on this process (Table 1).^45^^,^^61^ This process is also accompanied by the release of anti-inflammatory mediators, including IL-10, TGF-β, PGE2, hepatocyte growth factor (HGF),^45^ and IL-4^21^ (Fig. 3).

## The role of efferocytosis-related cells in wound repair

**Figure 4:**
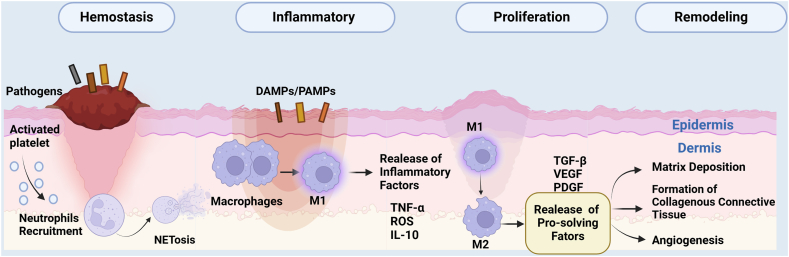
Phases of wound healing and the roles of cell-mediated efferocytosis in tissue regeneration. First and foremost, upon tissue injury, blood clots immediately form to temporarily plug the wound with platelets. Soon after this, the inflammatory response of the wound occurs through the migration of neutrophils drawn from local resident populations into the wound. Neutrophils may release reactive oxygen species (ROS), proteases, proinflammatory mediators, pro-resolving mediators, and neutrophil extracellular traps (NETs), which are useful for macrophage recruitment and polarization. Moreover, in concordance with M2 polarization, efferocytosis is enhanced. These are the hemostasis and inflammation phases. Next, resurfacing is driven by re-epithelialization, which includes epidermal migration, matrix deposition, and angiogenesis via fibroblasts. Finally, remodeling the extracellular matrix is the last stage resulting in tissue repair.

Acute or physiologic inflammation triggered by tissue damage is an essential body's first line of defense to maintain homeostasis, but it must be properly completed by a mechanism termed “efferocytosis”.^22^ Above, we already know about the various cells involved in the efferocytosis process, including professional and amateur phagocytes. Next, we will introduce the role of efferocytosis in wound repair at the cellular level. Tissue damage immediately triggers the inflammatory response through four well-orchestrated stages of hemostasis, inflammation, proliferation, and remodeling^66^^,^^67^ (Fig. 4). Incomplete efferocytosis in any of these four processes leads to nonhealing of the wound, which is largely observed in diabetic wounds.^66^ Prolonged low-grade inflammation is believed to result in chronic wounds characterized by a constant influx of neutrophils, a lack of anti-inflammatory macrophages, impaired angiogenesis, and elevated matrix metalloprotease levels.^9^^,^^68^ Unsurprisingly, the immune dysregulation that occurs as a consequence of aging or conditions such as diabetes has a substantial negative effect on tissue healing and is often characterized by persistent inflammation at the injured site because of defective efferocytosis. We will state the cells and signals, such as the release of chemokines, recruitment of neutrophils and M1 macrophages, the switch to M2 macrophages, and finally angiogenesis, as well as collagen deposition.

## Platelets and endothelial cells

After tissue damage, the most urgent requirement for platelets is hemostasis, including intrinsic and extrinsic coagulation cascade pathways that stop blood loss and cause blood clots to close wounds.^69^

During this process, platelets secrete three major types of granules, namely, α-granules, dense granules (or δ-granules), and lysosomes.^70^ Moreover, α-granules contain various kinds of proteins that release cytokines, chemokines, and adhesive molecules for immune cells, such as neutrophil recruitment into the injury area, which can contribute to the removal of debris and can also help to activate more platelets to support coagulation.^70^ Finally, these factors will be introduced in this review.

## Neutrophils

The current consensus is that neutrophils are the first immune cells recruited to the wound within 12–24 h after injury and make up ∼50% of all cells. The neutrophil recruitment process starts at the interface between hemopoietic cells and endothelial cells and involves several steps, in which neutrophils undergo slow rolling (neutrophils are propelled by blood flow and can roll on inflamed endothelium via selectin, integrin, and their ligands^71^). Additionally, the adhesion of these cells is augmented through integrin-mediated interactions, intraluminal crawling, as well as paracellular and transcellular migration. As the molecular mechanisms underlying each step are being gradually elucidated, it has become evident that the expression of chemokines on the abluminal surface of the endothelium is crucial for the arrest of rolling neutrophils before their transmigration.^72^^,^^73^ Neutrophils display a wide range of effector mechanisms to counteract pathogens in wounds, including phagocytosis, the release of reactive oxygen species (ROS), proteases, the generation of proinflammatory mediators and pro-resolving mediators, and neutrophil extracellular traps (NETs).^74^^,^^75^ Here, we focused on the role of NETs in efferocytosis and wound healing. Locally activated neutrophils initiate increased recruitment of immune cells via autocrine and paracrine signaling via chemokines, cytokines, and chemoattractants, termed “neutrophil swarming”.^76^ Moreover, neutrophils secrete proteases that amplify platelet responses by activating protease-activator receptors and by splitting cells into cell debris.^77^ These effects are a double-edged sword, on the one hand, causing severe collateral damage to tissues and, on the other hand, promoting wound repair via proinflammatory mediators against invasive microorganisms.^9^^,^^16^ In the inflammatory phase, infiltration of the wound site at the early inflammatory phase inhibits microbe particle invasion and growth in the host via phagocytosis, proinflammatory mediators, and the generation of cellular oxidative species such as ROS. Hence, the microenvironment of injured tissues can be suitable for the progression of proliferation and further remodeling.^72^^,^^74^ In the third section, we will introduce in detail how neutrophil-mediated efferocytosis affects the underlying molecular mechanisms involved in wound repair. Since neutrophils are so useful, is it truly good to have them all the time? The answer is most obviously no. Their persistence has been associated with aggravated and prolonged tissue damage, leading to chronic and non-resolving inflammation. Neutrophil engulfment, known as efferocytosis, is a reparative process for healing.^78^ During inflammation observed in zebrafish, neutrophils and macrophages were tracked individually, and x and y coordinates were plotted, which demonstrated that neutrophils strongly undergo reverse transendothelial migration via the identification of a neutrophil meandering index dependent on phagocytosis, known as efferocytosis.^71^ This process results in the return of neutrophils to the bone marrow mediated by C-X-C chemokine receptor type 4 (CXCR4).^78^ Reverse migration may not occur in acute respiratory distress syndrome because neutrophils are likely to stay in the wound to persistently damage lung tissue.^79^ Neutrophil efferocytosis and reverse migration not only play direct roles in wound repair but also play indirect roles via macrophages.

## Macrophages

MΦs, which originate from mononuclear myeloid lineage cells, are critical immune cells that participate in efferocytosis *in vivo*.^41^^,^^46^^,^^80^ Macrophages reside in different kinds of tissues under homeostatic conditions and are derived from monocytic precursors that infiltrate the injured wound.^21^ Normally, macrophages are dichotomously classified into classically activated M1 and alternatively activated M2 phenotypes.^81^ Dal-Secco and colleagues found that proinflammatory CCR2^hi^ CXC3CR1^lo^ monocytes are recruited early and persist for at least 48 h following sterile injury. As a result, these cells transition *in situ* to pro-resolving CCR2^lo^ CXC3CR1^hi^ macrophages, which turn into the injury site and are essential for efficient tissue repair.^82^ In people with diabetes, macrophages exhibit hyperresponsiveness to inflammatory stimulants and increased secretion of proinflammatory cytokines rather than an increased ability to phagocytose pathogens through efferocytosis.^83^ These M1 macrophages have an elevated capacity to destroy and phagocytose microorganisms and corpses to maintain a clean area of injury in the inflammatory stage.^82^ These cells can detect and respond to a variety of pathogens and environmental stimuli through the release of cytokines, chemokines, growth factors, and oxygen-derived free radicals, such as TNF-α/IL-6/inducible nitric oxide synthase (INOS)/nitric oxide (NO), and this process has been shown to cause cardiac damage.^75^^,^^84^ Moreover, the study showed that M2 macrophages could accelerate the proliferation and migration of fibroblasts in a paracrine manner in the remodeling phase.^85^ In the phase of proliferation and remodeling, the switch from M1 macrophages to M2 macrophages means the switch from proinflammatory to anti-inflammatory.^86^

## Dendritic cells

Apoptotic cell uptake (efferocytosis) by dendritic cells has been linked mainly to their antigen presentation properties and phagocytosis ability, which are similar to those of macrophages.^87^ These cells have been phenotypically classified as classical dendritic cells or plasmacytoid dendritic cells based on cell surface markers and functional criteria.^88^ In response to stimuli, plasmacytoid dendritic cells are recruited by C-X-C motif chemokine ligand 10 (CXCL10) released by neutrophils, which accelerates repair via stimulation of fibroblast and macrophage growth factor responses.^89^ C-type lectin domain-containing 9A (Clec9A), which can recognize DAMPs, is expressed on the surface of resident dendritic cell subsets specialized for downstream signals, such as the remodeling of the cytoskeleton with particular actin-binding domains.^90^ It has been proven that dendritic cells distinguish LPS in bacteria through Toll-like receptors (TLRs)^91^ and distinguish viruses through surface receptors such as TLR3.^92^ Both classical dendritic cells and plasmacytoid dendritic cells respond to host-derived nucleic acids during injury through TLR7-and TLR9-dependent pathways.^93^ These findings reveal a new role for dendritic cells in sensing tissue damage and reparative processes for healing at skin surfaces. The abovementioned TLRs participate in efferocytosis through the “find-me” signal, suggesting that efferocytosis is connected with the function of dendritic cells in wound repair.^8^

## Fibroblasts

The proliferation and remodeling phase is distinguished by the infiltration of diverse cellular components. During this stage, matrix deposition takes place, along with the formation of collagenous connective tissue and angiogenesis. Fibroblasts, which are cells within the body's connective tissue, play a pivotal role. They are responsible for generating and remodeling the extracellular matrix. This activity contributes to the restoration of the tissue barrier. Additionally, if efferocytosis occurs, it can also lead to scar formation.

## The role of signals and factors in efferocytosis during tissue repair

### Release of chemokines in platelets and endothelial cells

In a tissue injury model, distinct outer chemoattractant zones are formed by activated platelets. The DAMPs released by injured necrotic cells stimulate tissue-resident cells to secrete interleukins, such as IL-1, which up-regulate chemokines involved in intravascular intercellular adhesion molecule 1 (ICAM-1) and intravascular gradients of the CXCR2 ligands CXCL1 and CXCL2 in the outer zone.^72^ Blockade of chemokine receptors such as chemokine receptor 2 (CCR2) and CCR5 seems to be a more feasible strategy for blocking cell–cell communication via macrophages. The CCR2/5 antagonist cenicriviroc improved macrophage recruitment in mouse models of NASH, which confirmed the role of chemokines in efferocytosis-mediated wound repair.^94^ Moreover, CCR1, CCR2, CCR5, and CXCR2 mainly act on the recruitment of inflammatory cells in larger arteries, indicating the influence of chemokines on heart repair after atherosclerosis.^16^ De Juan et al showed that endothelial cell-specific Bmal1 depletion disrupted time-of-day-dependent leucocyte adhesion in veins via the expression of ICAM-1 and vascular cell adhesion molecule 1 (VCAM-1) on vascular beds.^16^ Although they promote inflammation to inhibit pathogens, these chemokines, released by endothelial cells, need to be cleared because they regulate the recruitment of neutrophils to suppress tissue repair. Mechanistically, Dean and colleagues determined that MMP-12 specifically cleaves the partial motif of CXC chemokines (CXCL1, CXCL2, CXCL3, CXCL5, and CXCL8) to inhibit the ability to activate more neutrophils and macrophages.^95^ These chemokines all play their important part in efferocytosis during wound repair.

### NETs in neutrophils

The chemotaxis of neutrophils toward wounds is regulated by a wide variety of receptors, such as DAMPs and PAMPs, colony-stimulating factor (G-CSF), platelet-derived growth factor (PDGF), and vascular endothelial growth factor (VEGF).^72^^,^^96^ As mentioned above, we focused on the role of NETs released during efferocytosis in wound closure. Taking advantage of the depolymerized DNA structure as their skeleton, NETs, a weblike structure stimulated by phorbol myristate acetate, contain histones, myeloperoxidase, cathepsin G, and other bactericidal and proinflammatory mediators released into the extracellular space.^74^^,^^78^ NETs promote the inhibition of pathogens such as *E*. *coli* in acute inflammation, but they also lead to endothelial cell damage and organ injury via the N-terminal histone tail. Moreover, the persistent existence of NETs may result in several chronic inflammatory diseases, including chronic obstructive pulmonary disease and diabetes.^78^ The chemokine IL-1 released from NETs can enhance vascular obstruction and delay heart repair in atherosclerosis.^97^ The levels of NET components, such as elastase, are elevated in the blood of patients with diabetic foot ulcers, and Fadini and colleagues first demonstrated that NETs inhibited the process of diabetic wound healing.^98^ Therefore, NETs clearance is crucial for removing acute and chronic inflammation to promote wound healing. The most obvious evidence is that neutrophils usually only have a 24-h lifespan.^11^ Neutrophils that swarm inflammation sites undergo lifespan loss, and cytokines are released by phagocytic macrophages. It is speculated that CX3CL1 and its receptor CX3CR1-mediated efferocytosis are arguably impactful for NETs clearance, preventing further leukocyte recruitment and resolving the exudate.^72^

Moreover, it is surprising that neutrophils can also play an important role in the switch of macrophages. The uptake of apoptotic polymorphonuclear PMNs by macrophages is also termed efferocytosis. Neutrophilic recruitment is a prerequisite for the inflammatory-to-proliferative transition of macrophages, which is necessary for quality wound repair.^72^

### Signals and factors involved in the recruitment and switching of macrophages

As reviewed above, at different stages of wound healing, macrophages are divided into different phenotypes and play different roles. In the early stages of wound healing, a characteristic proinflammatory phenotype named M1 is observed. Depending on the local environment, macrophages exhibit an appropriate phenotype to execute their specialized tasks. Classically activated M1 macrophages are involved in defense against bacteria and viruses, acting as “find-me” signal.^38^ For example, the secretion of IL-12, IL-23, TNF-α, IL-6, INOS, and NO promotes the secretion of interferon-γ by macrophages in response to LPS.^21^^,^^99^

However, when efferocytosis of the M1 phenotype is not exhausted, it may persist for a long time. It has been confirmed that M1 macrophages are involved in the whole process of the complex development of atherosclerosis, including its formation, progression, and eventual rupture.^100^ A lack of TGF-β1 signaling, a mediator in the “engulfment and digestion” stage, in Smad3 knockout mice leads to a significant decrease in inflammatory M1-polarized macrophages at the wound site.^101^ Moreover, the IL-4-activated signal transducer and activator of transcription 6 (STAT6) transcription factor is required for direct M1 phenotype suppression both *in vitro* and *in vivo*, which builds another polarization-specific epigenomic signature to reduce inflammation during wound repair.^102^

Several anti-inflammatory M2-activated signaling cascades have been proven to be aimed at suppressing the M1 phenotype and enhancing the M2 phenotype. Accumulating evidence suggests that when the switch from M1 phagocytes to M2 macrophages is impaired, wound healing stalls in the inflammatory phase, and a chronic wound occurs.^82^ Notably, the mutual effect between apoptotic cells and macrophages results in the formation of apoptotic cell degradation products, and their metabolites, as well as other cytokines, prompt intracellular signaling cascades that in turn boost the switch from the M1 to M2 phenotype in cell reprogramming.^85^^,^^103^ M2 macrophages express CD206, a mannose receptor,^104^ and can be marked by cluster of differentiation 163 (Cd163), Arginase-1 (Arg1), YM1 (chitinase-like 3, Chil3), and TGF-β1, which can be detected to indicate the M2 phenotype.^105^

**Figure 5:**
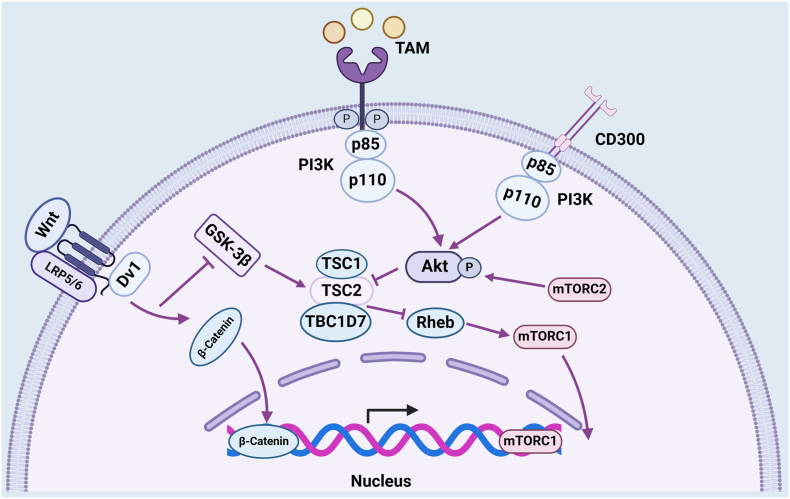
Role of PI3K-AKT signaling in macrophage polarization. PI3K-AKT can connect “eat-me” stage signals, such as CD300b and MerTK, with wound repair. PI3K-AKT can activate the TAM receptor via the p85 regulatory subunit through the suppression of GSK-3β. The reduction in GSK-3β is related to the Wnt/β-catenin pathway, which is beneficial for regeneration.

The phosphoinositide 3-kinase (PI3K)/protein kinase B (AKT) pathway is a major intracellular signaling pathway that contributes to the regulation of apoptosis via the transformation of the macrophage phenotype. The PI3K/AKT signaling pathway serves as an exciting target for the transition to M2, which can bind to the TAM receptor via its intracellular kinase domain 38. PI3K is composed of a p85 regulatory subunit and a p110 catalytic subunit. TAM receptors are considered to bind with p85 through growth factor receptor-bound protein 2 (Grb2). Activated PI3K then phosphorylates AKT and suppresses the glycogen synthase kinase-3β (GSK-3β) axis.^38^ GSK-3β also affects other signals in wound repair. Yin et al demonstrate that the Wnt/β-catenin pathway arguably impacts liver regeneration by reducing GSK-3β phosphorylation, which affects the Wnt/β-catenin pathway, thereby inhibiting liver regeneration accompanied by the polarization of M1 macrophages.^106^ Wnt signaling pathways are known to promote wound closure and keratinocyte migration.^43^ The connection between Wnt ligands and frizzled receptors promotes the translocation of the downstream molecule β-catenin from the cytoplasm to the nucleus to up-regulate gene transcription.^107^ Recently, it has been proven in mouse models that Wnt4 increases the thickness of the epidermis in burn wounds and activates classic Wnt/β-catenin signaling to promote the migration of epidermal cells by inhibiting cell adhesion molecules such as integrin alpha 6 (ITGα6), integrin beta 1 (ITGβ1), and E-cadherin 108. Although it has not yet been studied in human samples, it still provides an idea to promote wound healing. In patients with inflammatory bowel disease, activated nuclear β-catenin, in parallel to phosphorylated mTOR, suggests that Wnt1 activates mTOR through the inhibition of GSK-3β. The proportion of Wnt1-expressing macrophages is greater in damaged mucosa than in normal tissue, which supports the role of Wnt signaling in efferocytosis and polarization.^99^ In summary, the Wnt signal serves as an exciting target for wound closure. Accumulating evidence suggests that intradermal injection of IL-25 into full-thickness wounds promotes M2 macrophage polarization via PI3K-AKT signaling.^80^ Murakami et al also advanced our understanding of the mechanism by which CD300b acts as an “eat-me” signal in the PI3K/AKT signaling pathway. They silenced CD300b expression and found diminished PI3K-Akt kinase activation and impaired efferocytosis.^36^ Thus, PI3K-AKT has been proven to suppress complications associated with diabetic wounds. In addition to affecting TAMs and the GSK3β axis, PI3k-AKT also targets other downstream signals or is regulated by upstream signals. A recently identified macrophage TLR, a mediator in the “find-me” signal, serves as an exciting target for the B-cell adapter for PI3K (BCAP). Moreover, mice deficient in BCAP exhibit prolonged intestinal inflammation and prolonged reparative processes for healing.^109^ NADPH oxidase 2 (NOX2) is expressed in inflammatory cells (macrophages and neutrophils) and resides in a variety of tissues throughout the body, including the brain, neurons, microglia, heart, and kidney.^110^ NOX2-deleted alveolar macrophages cause heightened lung inflammation, as macrophages are activated to maintain pulmonary homeostasis.^111^ In parallel, mice lacking NADPH oxidase were found to be prone to acute hyperinflammatory reactions, exhibiting increased inflammatory cell infiltrates.^75^ Therefore, PI3K-AKT signaling serves as an exciting target for regulating phenotype switching via NOX2. Notably, the NOX2-specific inhibitor GSK2795039 significantly prevented plaque vulnerability and decreased intraplaque hemorrhage in atherosclerosis patients. There are a few known roles of NOX2 in vulnerable plaque pathogenesis, and NOX2-ROS-MerTK is involved in regulating macrophage polarization-mediated efferocytosis (Fig. 5).^112^ These may provide evidence that NOX2 in the PI3K/AKT pathway contributes to cardiac repair in atherosclerosis.

Apart from the PI3K-AKT pathway, nuclear receptors (NRs) can be associated with efferocytosis through M2 phenotype transition.^39^ They are a group of transcription factors that are widely expressed in the body and can be expressed in the cytoplasm or nucleus.^113^ Members of the NR subfamily 4 group A (NR4A or Nur77) subgroup, which is activated by many stimuli, promote apoptotic signals. Research has demonstrated that knocking down Nur77 reduces cellular proliferation and angiogenesis.^114^ Thomas et al studied the retention of nuclear receptor subfamily 4 group A member 1 (Nr4a1) gene expression in macrophages treated with LPS, which switched from inflammatory lymphocyte antigen 6C (Ly-6C) (high) to reparative Ly-6C (low) monocytes/macrophages (Mo/MΦ).^115^ This process, related to S100A9 acting as an “eat-me” signal in efferocytosis, has been proven in mice both *in vivo* and *in vitro* through the detection of Nur77 expression via S100A9 blockade.^116^ NF-κB is a major transcription factor associated with the expression of a wide range of proteins and related genes that mediate inflammation and immune responses.^117^ Activated Notch1 signaling occurs in diabetic wounds because of high glucose levels via the Dll4–Notch1 axis and a positive feedback loop.^118^ Moreover, emerging evidence indicates that aberrant activation of Notch signaling is connected with decreased M2 activation and function and low M1 gene expression.^85^^,^^119^ Further study suggests that DII4 is highly expressed in response to stimuli, including LPS and TLR4, which initiates the activation of the NF-κB pathway via Notch1 signaling. This process has been found in systemic lupus erythematosus patients with disorders of inflammation.^119^ Notably, Notch promotes forkhead box O1 (FoxO1) activation at gluconeogenic promoters, leading to glucose intolerance, which may partially explain the mechanism of diabetes.^94^ A comprehensive study related to the nuclear factor erythroid 2-related factor 2 (Nrf2) transcription factor suggested that Nrf2 overexpression promoted autophagic flux, inhibited M1 phenotype polarization, and promoted M2 phenotype polarization, which prevented sepsis-induced lung injury and inflammation in an Nrf2 knockout mouse model.^120^ A study also showed that Nrf2 promotes M2 polarization partly through inhibiting NF-κB and activating PPAR-γ.^120^ Nrf2 initially acts as a master regulator of antioxidants through the regulation of ROS levels, which play a pivotal role in cellular defense against inflammation.^22^^,^^121^ In skeletal muscle regeneration, increased expression of the transcription factor Nfix (a member of the nuclear factor I (Nfi) family) can promote an anti-inflammatory mechanism and reverse the macrophage phenotype by inhibiting the RhoA-ROCK1 pathway, which is essential in the digestion phase of efferocytosis.^122^ Following the engulfment of apoptotic cells, nuclear receptors are also engaged in M2 polarization via the transcription of relevant genes. Specifically, efficient clearance of apoptotic cells has been demonstrated through the activation of LXRs, RXRs, and PPAR-γ.^38^^,^^122^ Hong et al demonstrated that LXRs contribute to the control of neutrophil engulfment by macrophages. Using gain- and loss-of-function mouse models, they found that LXR signaling regulated this process in a Mer-dependent manner and suppressed the IL-23/IL-17/G-CSF cytokine cascade.^123^ Moreover, in the absence of PPAR-γ, mouse macrophages exhibit increased proinflammatory cytokine levels as well as the M1 phenotype and decreased anti-inflammatory cytokine levels as well as the M2 phenotype when stimulated with LPS.^124^ Chen et al demonstrated that PPAR-γ plays a pivotal role in controlling wound macrophage polarization to ensure efficient skin wound healing by using PPAR-γ-knockout (KO) mice, which exhibit delayed wound closure.^125^ In support of the relevance of PPAR-γ in apoptotic cell phagocytosis by macrophages, Yoon and colleagues reported decreased efferocytosis in lung fibrosis with GW9662, a kind of PPAR-γ antagonist.^126^

### Signals and factors involved in angiogenesis and collagen deposition

Tissue injury is usually accompanied by hemorrhage in the first stage and matrix degradation and remodeling in the last phase. To promote wound closure, angiogenesis must be promoted. The primary proangiogenic factor during tissue healing is VEGFA.^96^ This conclusion has been verified in a zebrafish model.^127^ *In vitro* research revealed that blocking vascular endothelial growth factor receptor (VEGFR) on endothelial cells only partially (15%) impaired the proliferation of endothelial cells activated by thrombin. Other important factors, including pigment epithelium-derived factor (PEDF), insulin-like growth factor 1 (IGF-1), TGF-β, and the basic fibroblast growth factor 2 (FGF2) derived from efferocytosis, serve as targets for angiogenesis, collagen deposition, and tissue repair.^86^^,^^128^ Farooq and colleagues reported that the FGF-FGFR signaling pathway can mediate the development of multicellular organisms and promote angiogenesis and regeneration in adults.^128^ As comprehensively reviewed by Ornitz and Itoh, the regulation of FGF signaling can also be controlled by PI3k/AKT, which plays an important role in the M2-like phenotype during efferocytosis.^129^ Phospho-FGFR phosphorylates the docking protein FGFR substrate 2 (FRS2). Activated FRS2*α* binds the membrane-anchored adaptor protein growth factor receptor-bound 2 (GRB2) and the tyrosine phosphatase SHP2. Finally, GRB2 activates the PI3K-AKT pathway via the recruitment of GRB2-associated binding protein 1 (GAB1).^130^ PEDF inhibits angiogenesis and healing by decreasing the expression of VEGFR2 and VCAM-1.^131^ TGF-β1 improves the angiogenic ability of endothelial progenitor cells and endothelial colony-forming cells *in vitro* because it increases endothelial colony-forming cell viability and migration.^101^^,^^132^ The profibrogenic effects of TGF-β are mediated by SMAD-dependent pathways initiated by the recruitment and phosphorylation of Smad2 and Smad3.^67^^,^^94^ Moreover, TGF-β1 also provides mechanistic insight into myofibroblasts, another kind of cell characterized by their expression of α-smooth muscle actin, which acts on the remodeling phase of efferocytosis.^133^ For example, TGF-β1 promotes the conversion of a smooth muscle actin (a-SMA)-negative fibroblasts to a-SMA-positive myofibroblasts, greatly increasing collagen synthesis and contractile force.^133^

## Therapeutic applications and potential targets of efferocytosis in wound healing

### “Find-me” signals

CX3CL1 plays a critical role in wound healing by recruiting macrophages to the injury site, modulating the inflammatory response, promoting angiogenesis, and facilitating tissue remodeling. Through these mechanisms, CX3CL1 enhances the clearance of pathogens, supports the transition from pro-inflammatory to anti-inflammatory phases, and accelerates tissue repair and regeneration.^134^ From a therapeutic standpoint, targeting CX3CL1 signaling presents a promising strategy for promoting wound healing, particularly in chronic or impaired healing contexts such as diabetic ulcers. However, excessive CX3CL1-mediated inflammation may aggravate tissue damage. So, future therapeutic methods could place emphasis on controlling CX3CL1 activities or combine it with other modulators of macrophage polarization to optimize its regenerative effects.

### “Eat-me” signals

The gene encoding normal Mertk was introduced into macrophages using vectors (such as adeno-associated virus). In a mouse model of atherosclerosis, the injection of adeno-associated virus carrying the Mertk gene significantly enhanced the phagocytosis of apoptotic cells by macrophages, reduced the accumulation of apoptotic cells in plaques, and increased plaque stability.^135^

Platelet-related receptors, ADP receptors (P2Y1 and P2Y12), and cyclooxygenase can be used as efferocytosis-related therapeutic targets, with potential to improve abnormal efferocytosis in atherosclerosis by regulating platelet–monocyte interactions and related processes.^136^ Targeting platelet receptors like P2Y12 offers a promising approach to enhance efferocytosis in atherosclerosis by modulating platelet–monocyte interactions. However, clinical translation may be limited by bleeding risks and inflammatory complexity.

Plasma extracellular vesicles can suppress the inflammatory reaction to PAMPs by reducing IL-6 and TNF-α and increasing IL-10 secretion. What's more, extracellular vesicles enhance the transition of macrophages from M1 to M2. All these activities in efferocytosis promote wound repair.^137^ The ability of plasma extracellular vesicles to orchestrate a shift from pro-inflammatory to pro-resolving responses highlights their significant therapeutic potential, particularly in chronic wounds characterized by excessive inflammation. However, key challenges are related to the standardization of EV isolation, characterization, and dosing. Future clinical translation will depend on solving these puzzles.

## Conclusions

As shown in this review, the capacity for efferocytosis is enormous. In contrast to the rapid resolution of dead cells, there are various kinds of diseases, including atherosclerosis, diabetes, and inflammatory bowel disease, for which the quantities of uncleared apoptotic cells are recruited to the injured area. Therefore, therapeutic strategies aimed at promoting efferocytosis to suppress inflammation and promote wound closure have been studied.^138^^,^^139^ In addition to this process, there is still great potential to develop other approaches for treating diseases owing to excessive apoptosis, inefficient efferocytosis, and impaired repair.

## CRediT authorship contribution statement

**Yilin Sun:** Writing – review & editing, Writing – original draft, Validation, Investigation, Data curation. **Haiying Guo:** Writing – review & editing. **Yang Bai:** Writing – review & editing, Funding acquisition, Data curation. **Jin Chen:** Writing – review & editing, Supervision, Funding acquisition, Conceptualization. **Yuhong Li:** Writing – review & editing, Funding acquisition, Conceptualization.

## Funding

This work was supported by the 10.13039/501100001809National Natural Science Foundation of China (No. 82173446, 82201298), the 10.13039/501100005230Natural Science Foundation of Chongqing Municipality (China) (No. CSTB2025NSCQ-GPX0626, CSTB2022NSCQ-MSX0162), and the Key Research and Development Projects of Hainan Province of China (No. ZDYF2024SHFZ062).

## Conflict of interests

Yuhong Li is an Editorial Board member of *Genes & Diseases*, but he has no involvement in the peer-review of this article and has no access to information regarding its peer-review. The rest authors declared no competing interests.
